# 2413. Drug Use Associated vs Non-Drug Use Associated Endocarditis in an Academic Center from 2012-2017

**DOI:** 10.1093/ofid/ofad500.2033

**Published:** 2023-11-27

**Authors:** Mohammad Mahdee Sobhanie, Meagan J Clark, Courtney Hebert, Michael Haden

**Affiliations:** The Ohio State University, Columbua, Ohio; The Ohio State University, Columbua, Ohio; The Ohio State University, Columbua, Ohio; The University of Colorado, Denver, Colorado

## Abstract

**Background:**

Drug use associated Infective Endocarditis (DU-IE) has been noted to be increasing over the last decade(Clinical Infectious Diseases® 2020;71(7):1664–7). In this study we sought to assess the unique aspects of DU-IE compared to IE not associated with drug use. We undertook an in-depth descriptive study of patients who presented with IE over the course of 5 years at one large academic medical center.

Table 1

**Figure:**
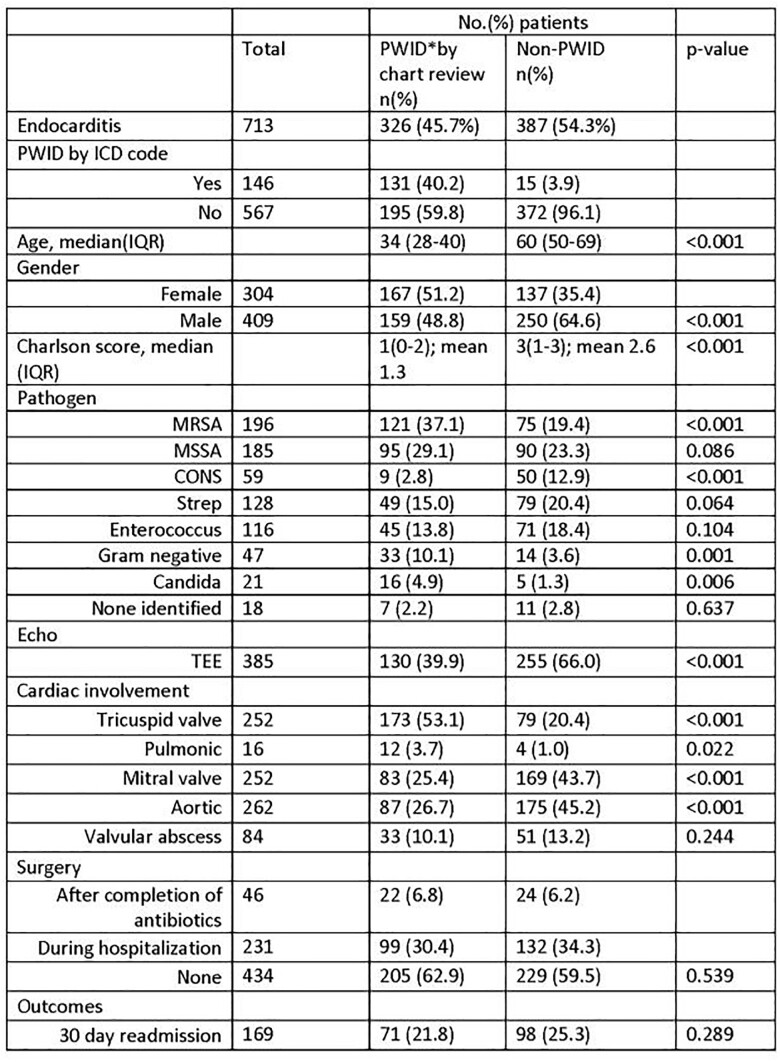

**Methods:**

This was a retrospective chart review of patients with IE admitted to the Ohio State Wexner Medical center between 2013 and 2017. Prisoners, patients < 18 or > 89 years of age, and pregnant patients were excluded from the study. The first admission during the study period in those with definitive or probable endocarditis by Duke criteria, were included. Persons who inject drugs (PWID) with IE were compared with those with IE without documentation of drug use. DU was determined by both ICD coding and manual chart review. Given the discrepancy between the two, chart review was used for the analysis. Differences in proportion were compared using Fisher’s exact, and continuous variables using student’s t-test.

**Results:**

Overall, 713 patients met inclusion criteria. Approximately half (45.6%) of the patients had DU-IE, this proportion generally increased over the 5 years, with 59.8% of the cases being DU-IE in 2017. DU-IE patients were more likely to be younger, female, and have a lower Charlson comorbidity score. DU-IE patients were significantly more likely to have MRSA, Gram Negatives and Candida species, whereas non DU-IE was more likely to have Coagulase negative staph species. DU-IE patients were less likely to have a transesophageal echocardiogram, and unsurprisingly were more likely to have tricuspid valve involvement, and pulmonary septic emboli.

**Conclusion:**

The patient population with DU-IE compared to non-DU IE differ from one another, and further studies are required to better understand long-term clinical outcomes in the treatment between each group.

**Disclosures:**

**All Authors**: No reported disclosures

